# The Temporal Evolution of Digital Learning: A Longitudinal Quasi‐Experimental Study of Web‐Based Versus Conventional SRH Education Among Adolescents

**DOI:** 10.1002/hsr2.73452

**Published:** 2026-10-27

**Authors:** Md Mizanur Rahman, Gerraint Gillan Ahi, Rafazila Ramli, Rasitasam Safii, Jacey Lynn Minoi, Stephanie Chua Hui Li, Ahmad Sofian bin Shminan, Lee Jun Choi

**Affiliations:** ^1^ Department of Community Medicine and Public Health, Faculty of Medicine and Health Sciences Universiti Malaysia Sarawak Kota Samarahan Malaysia; ^2^ Faculty of Computer Science and Information Technology Universiti Malaysia Sarawak Kota Samarahan Malaysia; ^3^ Faculty of Cognitive Sciences and Human Development Universiti Malaysia Sarawak Kota Samarahan Malaysia

**Keywords:** adolescents, digital intervention, indigenous, knowledge, sexual and reproductive health

## Abstract

**Background:**

Adolescents in rural indigenous communities often lack culturally appropriate sexual and reproductive health (SRH) education. This study compared the short‐term effectiveness of a web‐based SRH intervention versus conventional face‐to‐face education among 260 rural Bidayuh adolescents aged 16–19 in Sarawak.

**Methods:**

Using a non‐randomized, two‐district quasi‐experimental design (one district assigned to each arm to minimize contamination), knowledge was assessed via a validated questionnaire at baseline, 1 month, and 3 months. All 260 enrolled participants were analyzed under an intention‐to‐treat approach using a repeated‐measures ANCOVA (arm × gender × time); 15 participants (5.8%) were lost to follow‐up by 3 months.

**Results:**

Knowledge scores did not differ between arms at baseline or 1 month, but diverged significantly by 3 months, favoring the web‐based arm (68.12% vs. 59.13%; adjusted difference = −8.67 percentage points, 95% CI = [−12.61 to −4.74] (conventional minus web‐based; the web‐based arm scored higher), *p* < 0.001), reflected in a significant Arm × Time interaction, *F*(2, 747) = 7.47, *p* = 0.001. This advantage was numerically larger among females (*Δ* = −11.48, *p* < 0.001) than males (*Δ* = −5.87, *p* = 0.050), though the three‐way interaction did not reach conventional significance (*p* = 0.053). Sensitivity analyses (per‐protocol; multiple imputation) produced consistent estimates.

**Discussion:**

Knowledge retention over 3 months was better in the web‐based arm, with a numerically larger effect among females. These findings may be associated with digital SRH education promoting longer‐lasting knowledge among rural indigenous adolescents, but should be interpreted cautiously given the non‐randomized, two‐cluster design, which cannot fully separate intervention effects from district‐level differences.

**Conclusion:**

Self‐regulated digital platforms may be a promising addition to school‐ and community‐based SRH programs in conservative rural settings, alongside strategies to promote engagement and reinforcement, pending confirmation in a randomized, multi‐site trial.

## 1 Introduction

Adolescence is one of the most important phases in human development that is marked by fast physical, psychological, emotional, and social changes [1, 2]. Adolescents start puberty and sexual maturation during this time, which requires access to correct sexual and reproductive health (SRH) information to make informed health decisions [3]. Adolescents of reproductive age need extensive education on SRH; about 21 million adolescent girls in the developing world are exposed to unintended pregnancies every year, and young people between 15 and 24 years old or older bear a disproportionate burden of newly acquired sexually transmitted infections (STIs), such as HIV and HPV. Access to SRH information is a universal human right recognized by the United Nations, although this right is still not achieved in most cases with adolescents in marginalized and rural settings [2, 4].

Among indigenous and rural populations, the most pressing agenda for SRH education is the lack of geographical access and a socioeconomic deprivation crisis, further aggravated by a lack of educational infrastructure [5, 6]. Adolescents in these communities often mature without formal, personalized information about their bodies or rights because of widespread cultural and social taboos [7, 8]. Reproductive health is a secret or even prohibited issue in most conservative rural settings, including those in Southeast Asia and Africa, where families and educational institutions all remain silent or use a disjointed and faulty implementation of their curriculum [7, 8]. This insufficient information compels teenagers to use poorly informed peers or unproven media and develop misconceptions about risk and increased susceptibility to the ARH Triad of unwanted pregnancy, STIs, and substance abuse [3, 9, 10].

As much as the traditional face‐to‐face method is the conventional modality of school‐based sex education, there are major impediments to it in the conservative rural setting. Instructors in these areas do not always receive formal education, are personally uncomfortable when talking about sexual issues, or are afraid of backlash from religiously minded parents [1, 11, 12]. As a result, digital health interventions, such as web‐based courses, mobile applications, and gamification platforms, have become a promising alternative. Digital tools can be scalable and economical, where high‐quality and standardized content reaches remote populations without the need for the availability of skilled local facilitators [7, 13, 14]. More importantly, the cultural stigma and embarrassment around SRH issues will be alleviated by the anonymity and privacy offered by digital platforms, thereby helping create a more open learning environment [2, 15, 16].

Nevertheless, there is a notable issue: although digital means are more effective, their long‐term efficacy is under‐investigated, especially in conservative cultural environments [2, 17]. Most of the current literature focuses on the immediate outcomes of a post‐test, without considering the temporality of the learning process and the consolidation or forgetting of knowledge [18, 19]. Numerous digital interventions have positive short‐term effects on knowledge and attitudes that dissipate within 6 months, indicating a catastrophic lapse in information without reinforcement [2, 20]. Moreover, the evidence from rigorous, randomized controlled trials (RCTs) that compare the longitudinal trajectories of web‐based and face‐to‐face delivery in indigenous cohorts that have to manage the process of intense cultural surveillance is also quite a gap [14, 21, 22].

This study addresses these gaps by evaluating the effectiveness of a web‐based mobile intervention compared to conventional classroom delivery among a cohort of indigenous Bidayuh adolescents in rural Sarawak. This research is significant because its longitudinal design examines learning outcomes at three time points: baseline, 1 month, and 3 months. By doing so, the study seeks to determine whether the privacy and self‐paced nature of digital learning facilitate better long‐term cognitive consolidation than the high‐intensity but often transient impact of traditional one‐shot interventions [1, 23]. Thus, the primary objectives of this study are to evaluate the longitudinal effectiveness of a web‐based SRH intervention compared with conventional face‐to‐face education on the knowledge and attitudes of rural indigenous adolescents, and to analyze the temporal evolution of learning by examining knowledge acquisition at 1 month and its retention or decay at 3 months.

## 2 Materials and Methods

### 2.1 Study Design

The study used a controlled longitudinal quasi‐experimental design with non‐randomized, district‐level allocation of intervention conditions to examine the comparative effectiveness of different SRH‐based educational modalities. The trial was registered with the ANZCTR (Trial number: FME/23/58), which is registered with the World Health Organization (WHO). The UNIMAS ethics committee granted ethical approval (Ref. No. FME/23/58). The research was structured into four consecutive steps: proposal preparation, ethical clearance, a pilot study, and actual implementation, culminating in the publication of results. A longitudinal design was used, with outcomes measured at baseline, 1 month, and 3 months to examine temporal variation in knowledge and behavior. This design and its reporting follow established recommendations for non‐randomized public health evaluations [24].

### 2.2 Participants

The study was conducted in the Kuching and Serian divisions of Sarawak, where two distinct districts were purposively selected to serve as the intervention and control sites to minimize contamination bias. A total of 260 indigenous Bidayuh adolescents were recruited as the primary study population. Inclusion criteria required participants to be aged 16–19 years, provide informed written consent, have parental consent, and possess an app‐supported mobile phone. Exclusion criteria applied to temporary guests, non‐residents, those with incompatible devices, and individuals with known mental health problems. A CONSORT flow diagram tracked recruitment, beginning with 469 assessed individuals and ending with 245 participants retained for the final 3‐month analysis (Figure 1).

**Figure 1:**
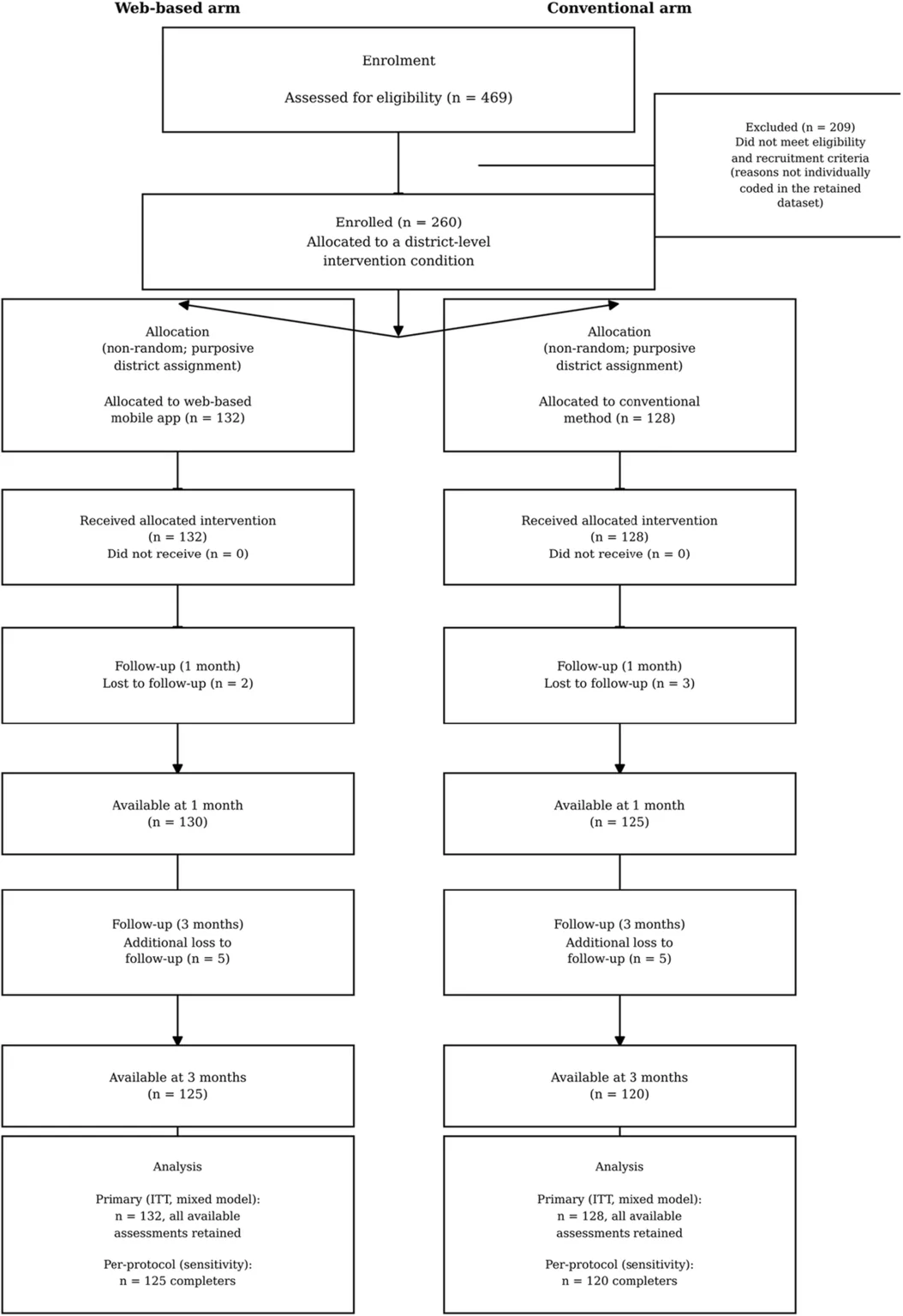
CONSORT flow diagram (participant‐flow diagram; non‐randomized, district‐level allocation, harmonized with Table 2 and Section 2.2).

### 2.3 Teaching Methods

A two‐arm intervention strategy was implemented to deliver SRH content through different channels.

#### 2.3.1 Web‐Based Teaching Approach

Participants exchanged feedback online, and providers delivered lessons via a shared connection. Those involved in this arm received the mobile app “RISES: Reproductive Information Strategies for Empowerment and Safety,” which includes four detailed modules. The app features interactive navigation, multimedia animated videos, quizzes, and a chatbot to facilitate engagement. Participants were trained to navigate and troubleshoot the app, allowing them to explore sensitive topics privately and at their own speed.

#### 2.3.2 Conventional Face‐to‐Face Approach

The same four SRH modules were delivered to the control arm through structured classroom teaching by trained PhD students. This mode was based on traditional lecture‐based instruction, group discussions, and printed resources provided in a physical educational institution.

### 2.4 Measures

A multidimensional questionnaire that comprised four main sections, including socio‐demographics; SRH knowledge; theory of planned behavior (TPB) constructs; and parent‐adolescent relationship, was used to gather data. The knowledge was evaluated in the four areas, including Sexual Growth and Development, Pregnancy and Abortion, Risky Sexual Behavior/STIs, and Parenting/Communication. Attitudes, subjective norms, control beliefs, perceived behavioral control, and behavioral intentions were psychological measures that were considered to be based on the TPB. The parent–child relationship scale measured closeness, communication, identification, adoration, and conflict/negative affect.

### 2.5 Module Development and Validation

The SRH modules were developed through a systematic process of adaptation and expert design, resulting in a sustainable intervention for adolescents. The research team conducted a thorough review and analysis of the available adolescent SRH modules in use by the Malaysian Ministry of Health, National Population and Family Development Board (LPPKN), Ministry of Education, and other international organizations like the WHO and the United Nations Population Fund (UNFPA). The new curriculum was designed to meet the needs of Malaysian young people and was structured into four modules: (1) Sexual Growth and Development; (2) Teenage Pregnancy and Abortion; (3) Sexual Risk Behavior, STIs, and Contraception; and (4) Parenting and Communication. Two doctoral researchers, a principal investigator, and a panel of experts from the Faculty of Medicine and Health Sciences helped develop the modules at Universiti Malaysia Sarawak. For the web‐based component, the Faculty of Computer Science and Information Technology created the RISES mobile application to deliver these modules through multimedia animated videos, interactive navigation, and chatbot services. Subject‐matter experts first validated all modules for content, language, and information technology before implementation. The mobile app was also rated individually across four dimensions, namely Engagement (interactivity), Functionality (performance/bugs), Esthetics (visual design), and Information Quality (accuracy and relevance). The app's reliability was demonstrated through psychometric analysis, with subscale Cronbach's Alpha values between 0.81 and 0.91.

### 2.6 Validation of Data Collection Instruments

Instruments to measure intervention effectiveness were validated in a four‐step procedure with high methodological strength and cultural suitability [25].

#### 2.6.1 Instrument Adaptation and Translation

The first step was defining constructs and creating items based on the TPB and available core instruments. The instrument was translated into Bahasa Melayu and then back‐translated by the linguistic specialists to ascertain accuracy in order to achieve linguistic and conceptual equivalence [13, 26].

#### 2.6.2 Content and Face Validity

Six adolescent health specialists were involved in content validation. The Item‐Level Content Validity Index (I‐CVI) and the Scale‐Level Content Validity Index (S‐CVI) had to be greater than 0.8. A two‐stage validation process was carried out, in which 34 items in the questionnaire (knowledge) were either changed or paraphrased until a majority of the constructs attained 1.00 perfect S‐CVI scores. A two‐way mixed‐effects model of inter‐rater agreement was used with 20 participants to determine face validity, which produced an excellent average measure of intraclass correlation coefficient (ICC) of 0.90 [27].

#### 2.6.3 Internal Consistency and Temporal Stability

Cronbach's alpha was used to assess reliability, with a value of over 0.70 considered acceptable. The total SRH knowledge scale had high internal consistency (0.924) across 42 items. Other constructs also met standard thresholds, including attitude (0.716), subjective norms (0.780), and behavioral intentions (0.839). Temporal stability was established using a 2‐week test–retest measure with 15 adolescents. The evidence showed that the majority of constructs, such as behavior change (*r* = 0.91) and SRH knowledge (*r* = 0.85), were strongly stable, indicating little measurement error over the period.

#### 2.6.4 Construct or Formative Analysis

To establish the construct validity of the Parent‐Adolescent Relationship Scale, an exploratory factor analysis (EFA) was conducted, and the solution was a five‐factor one. To prioritize theoretical consistency and psychometric sufficiency, the four‐domain structure of Closeness and Relationship Quality, Communication and Understanding, Identification and Admiration, and Value, Respect and Care was retained. This decision was supported by the fact that the omitted factor had low convergent validity, whereas the retained domains had better properties. Further validation included testing for Convergent and Discriminant Validity using Partial Least Squares Structural Equation Modeling (PLS‐SEM). Convergent validity was established because all constructs exceeded the Average Variance Extracted (AVE) threshold of 0.50 and exhibited Composite Reliability above the 0.70 criterion. Discriminant validity was determined using the Fornell–Larcker criterion and the heterotrait–monotrait (HTMT) ratio, and all findings were lower than the conservative 0.85 value. These rigorous analytical steps helped ensure that each dimension of the scale was empirically distinct and sufficiently differentiated.

### 2.7 Assessment of Instructional Method

The quality and fidelity of interventions were assessed through a systematic process evaluation. This was done using observer ratings (*N* = 20) and participant ratings (*N* = 260) to measure the delivered dose, received dose, fidelity, and reach of the training. The app was independently appraised based on engagement, functionality, esthetics, and information quality. Cronbach's alpha was used to determine psychometric quality. Assessment outcomes revealed high compliance with the modules to be implemented, and most sessions delivered 76%–100% of the intended content.

### 2.8 Data Analysis

Data were analyzed using IBM SPSS Statistics version 30.0 (IBM Corp., Armonk, NY, USA). The primary outcome was the percentage knowledge score at baseline, 1‐month, and 3‐month follow‐up. Group (web‐based, conventional) and gender (male, female) were treated as categorical variables. Age and baseline knowledge were used as covariates in the main models.

Baseline sociodemographic characteristics were compared between groups using chi‐square tests for categorical variables and independent‐samples *t*‐tests for age. Results are presented as counts and percentages for categorical variables, and mean and standard deviation (SD) for age. For chi‐square tests, *p* values and phi or Cramer's *V* were reported. At each time point, we used independent‐samples *t*‐tests to compare mean knowledge scores between the web‐based and conventional groups. For each comparison, we reported means, SDs, sample sizes, mean difference with 95% confidence interval (CI), Cohen's *d*, and the exact two‐sided *p* value. To examine change in knowledge over time and the effects of intervention arm and gender, we fitted a 2 (arm: web‐based vs. conventional) × 2 (gender: male vs. female) × 3 (time: baseline, 1‐month, 3‐month) repeated‐measures ANCOVA, estimated as a random‐intercept mixed model to account for correlation among repeated observations within participants. The model was fitted using a feasible generalized least‐squares (Swamy–Arora) random‐effects estimator, with denominator degrees of freedom based on observation‐level residual degrees of freedom (see Table 3 note for detail and caveats). Age was entered as a covariate. We reported *F*‐values, degrees of freedom, *p* values, partial eta squared, adjusted mean differences, and 95% CIs. The primary analysis followed an intention‐to‐treat approach: all 260 enrolled participants were analyzed according to their originally assigned condition, using every assessment each participant provided regardless of whether they completed all three waves. As a sensitivity analysis, missing values were multiply imputed using predictive mean matching (*m* = 20 imputations, pooled via Rubin's rules), and a per‐protocol (complete‐case) analysis restricted to participants completing follow‐up was also conducted for comparison. This approach follows recommendations for handling differential attrition in mobile health trials, where missingness is often not fully at random [28]. All tests were two‐sided with a significance level of 0.05.

### 2.9 Ethical Consideration

The research complied with all principles of the Declaration of Helsinki and obtained informed written consent from both adolescents and their legal guardians. Absolute confidentiality and anonymity were maintained, with participants provided with coded identifiers rather than names to ensure their privacy. Interviews were conducted in a private setting with no family members present to ensure respondents could speak openly about sensitive sexual behaviors. Each participant knew that withdrawing at any time was voluntary and that no negative consequences would be imposed. Research personnel were bound by a confidentiality agreement and received specialized training in human‐subjects protection.

## 3 Results

### 3.1 Characteristics of the Participants

In this study, 132 web‐based and 128 conventional participants were enrolled. The baseline characteristics of the participants (*N* = 260) showed no significant differences between the web‐based and conventional groups for sociodemographic variables. The mean age in the web‐based group was 17.18 years (SD = 1.145), compared to 17.02 years (SD = 0.996) in the conventional group, with an overall mean of 17.10 years (SD = 1.075; *p* = 0.060, *ϕ* = 0.169). Females comprised 56.2% of participants (*p* = 0.223, *ϕ* = 0.076), and the majority were single (98.8%; *p* = 0.596, *ϕ* = 0.063). Most participants had secondary education (73.5%), followed by pre‐university (12.3%) and no schooling (10.4%), with no significant group differences (*p* = 0.139, *ϕ* = 0.179). Overall, the two groups were comparable at baseline (Table 1).

**Table 1:** Baseline characteristics of the participants (*N* = 260).

Category	Total (%)	Web‐based (%)	Conventional (%)	*p*	Phi/Cramer *V*
Age in years					
16	39.2	40.2	38.3	0.060	0.169
17	25.4	18.9	32.0		
18	21.5	23.5	19.5		
19	13.8	17.4	10.2		
Gender					
Male	43.8	40.2	47.7	0.223	0.076
Female	56.2	59.8	52.3		
Marital status					
Single	98.8	99.2	98.4	0.596	0.063
Married	0.8	0.8	0.8		
Widowed	0.4	0.0	0.8		
Schooling status					
No schooling	10.4	12.1	8.6	0.139	0.179
Primary	0.8	0.8	0.8		
Secondary	73.5	70.5	76.6		
Pre‐university	12.3	15.2	9.4		
Religious school	1.2	1.5	0.8		
Others	1.9	0.0	3.9		

### 3.2 Changes in Knowledge in Different Follow‐Ups

At baseline, the two groups were well matched. Mean knowledge scores were almost identical (62.99% vs. 62.94%). The difference was not significant (*p* = 0.982), and the effect size was negligible (*d* = 0.003). At 1 month, scores moved slightly apart, but in the opposite direction. The web‐based group was slightly lower than the conventional group (61.20% vs. 63.73%). This difference was still not significant (*p* = 0.249). The effect size stayed small (*d* = −0.145). By 3 months, a clear gap had opened up. The web‐based group scored notably higher than the conventional group (68.12% vs. 59.13%), a difference of 8.99 percentage points (95% CI = [6.29–11.69], *p* < 0.001). Because variances were unequal at this time point, a Welch‐adjusted *t*‐test was used. It confirmed the result, *t*(197.70) = 6.57, *p* < 0.001. The effect size was large (*d* = 0.830) (Table 2).

**Table 2:** Comparison of mean percentage of knowledge scores between web‐based and conventional intervention groups across time points.

Intervention type	*n*	*M*	SD	*p*	Mean Diff	95% CI	Cohen's *d*
Baseline (Pre)							
Web‐based	132	62.99	18.50	0.982	0.05	[−4.36 to 4.47]	0.003
Conventional	128	62.94	17.64				
1 Month							
Web‐based	130	61.20	17.51	0.249	−2.53	[−6.83 to 1.78]	−0.145
Conventional	125	63.73	17.42				
3 Months							
Web‐based	125	68.12	13.25	< 0.001	8.99	[6.29–11.69]	0.830
Conventional	120	59.13	7.50				

*Note:* Means (*M*) and standard deviations (SDs) are reported for each group and time point. *t*(df) and *p* values are based on Levene's test for equality of variances (Welch's *t*‐test reported for 3‐month (Δ1 = 8.99; *t*(197.70) = 6.57, *p* < 0.001); equal variances assumed for baseline and 1‐month. Cohen's *d* is reported as the effect size.

### 3.3 Factorial Analysis for Knowledge Gain

To examine how SRH knowledge changed over time, and whether this change differed by intervention arm and gender, a 2 (intervention arm: web‐based vs. conventional) × 2 (gender: male vs. female) × 3 (time: baseline, 1‐month, 3‐month) repeated‐measures ANCOVA was conducted on knowledge score (%), controlling for age. We estimated the model as a random‐intercept mixed model to account for correlation among repeated observations within participants. Consistent with an intention‐to‐treat approach, we included all 260 enrolled participants and used every assessment each participant provided, regardless of whether they completed all three waves (760 person‐wave observations in total; 15 participants missing the 3‐month assessment and 5 missing the 1‐month assessment contributed their available data rather than being excluded).

There was a significant main effect of time, *F*(2, 747) = 9.41, *p* < 0.001, partial *η* ^2^ = 0.025, indicating that knowledge scores changed across the study period. The main effect of intervention arm was not significant, *F*(1, 747) = 1.67, *p* = 0.197, nor was the arm × gender interaction, *F*(1, 747) = 1.91, *p* = 0.167, indicating that, averaged across all three time points, overall knowledge level did not differ appreciably between arms. Gender had a significant main effect, *F*(1, 747) = 13.39, *p* < 0.001, partial *η* ^2^ = 0.018, with females scoring higher than males on average across time points and arms. Age was not a significant covariate, *F*(1, 747) = 1.12, *p* = 0.291.

Critically, the arm × time interaction was significant, *F*(2, 747) = 7.47, *p* = 0.001, partial *η* ^2^ = 0.020, indicating that the two arms followed different trajectories of knowledge change (Figure 2A). Simple‐effects tests at each time point showed no significant difference between arms at baseline (*Δ* = 3.78 percentage points, 95% CI = [−1.97 to 9.54], *p* = 0.197) or at 1 month (Δ = 1.86, 95% CI = [−3.89 to 7.61], *p* = 0.526), supporting adequate comparability between arms prior to the intervention taking full effect. By 3 months, however, the web‐based arm scored significantly higher than the conventional arm when averaged across gender (Δ = −8.67, 95% CI = [−12.61 to −4.74], *p* < 0.001; that is, the web‐based arm scored 8.67 percentage points higher).

**Figure 2:**
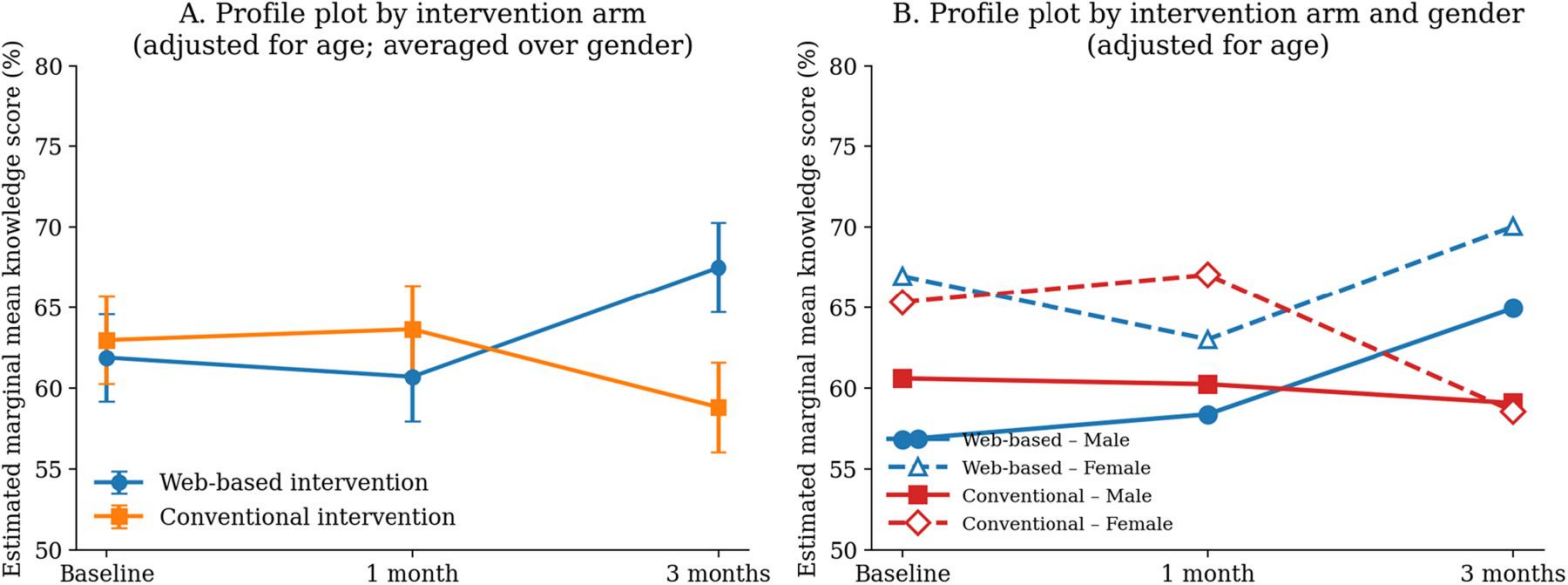
Profile plots of estimated marginal mean knowledge score (%) across time, adjusted for age, derived from the repeated‐measures ANCOVA in Table 3. (A) By intervention arm, averaged over gender. (B) By intervention arm and gender jointly. Error bars are 95% confidence intervals.

The three‐way arm × gender × time interaction approached, but did not reach, conventional statistical significance, *F*(2, 747) = 2.96, *p* = 0.053, partial *η* ^2^ = 0.008, as did the gender × time interaction, *F*(2, 747) = 2.92, *p* = 0.055, partial *η* ^2^ = 0.008. Descriptively, the 3‐month advantage of the web‐based arm was larger for females (Δ = −11.48, 95% CI = [−16.70 to −6.26], *p* < 0.001) than for males (Δ = −5.87, 95% CI = [−11.74 to 0.01], *p* = 0.050; Table 3, Figure 2). Because the three‐way interaction was only marginal, this apparent gender difference in the magnitude of the 3‐month intervention effect should be treated as a descriptive pattern warranting further investigation rather than a confirmed effect modifier.

**Table 3:** Repeated‐measures ANCOVA for knowledge gain by intervention arm, gender, and time.

Effect	df1	df2	*F*	*p*	Partial *η* ^2^	Δ [95% CI]
Omnibus effects						
Time	2	747	9.41	< 0.001	0.025	—
Arm	1	747	1.67	0.197	0.002	—
Gender	1	747	13.39	< 0.001	0.018	—
Arm × Time	2	747	7.47	0.001	0.020	—
Gender × Time	2	747	2.92	0.055	0.008	—
Arm × Gender	1	747	1.91	0.167	0.003	—
Arm × Gender × Time	2	747	2.96	0.053	0.008	—
Age (covariate)	1	747	1.12	0.291	0.001	—
Simple effects: Arm, by time						
Baseline	1	747	1.66	0.197	—	3.78 [−1.97 to 9.54]
1‐month	1	747	0.40	0.526	—	1.86 [−3.89 to 7.61]
3‐month	1	747	18.75	< 0.001	—	−8.67 [−12.61 to −4.74]
Simple effects: Arm at 3 months, by gender						
Males	1	747	3.84	0.050	—	−5.87 [−11.74 to 0.01]
Females	1	747	18.58	<;0.001	—	−11.48 [−16.70 to −6.26]

*Note: N* = 260 (760 person‐wave observations); analysis followed the intention‐to‐treat principle. Δ = adjusted mean difference in knowledge score (percentage points), conventional minus web‐based (simple effects by time) or as otherwise labeled; a negative Δ indicates the web‐based arm scored higher. CI, confidence interval. Model included a random participant intercept (variance = 98.93) with residual variance = 143.19 (intraclass correlation = 0.41), fitted via a feasible‐GLS random‐effects estimator for an unbalanced repeated‐measures design; denominator degrees of freedom (df2 = 747) reflect the observation‐level residual df of this estimator and are provided pending confirmation against REML‐based software (e.g., SPSS MIXED, R lme4) with Satterthwaite or Kenward–Roger degrees‐of‐freedom correction, which may adjust df2—particularly for the purely between‐participant effects (Arm, Gender, Arm × Gender, Age)—without changing the pattern of point estimates. Partial *η* ^2^ values are small in absolute terms, consistent with the modest proportion of variance any single factor explains in a three‐way repeated‐measures design with a covariate; effect sizes should be interpreted alongside the adjusted mean differences and their confidence intervals, not *p* values alone.

### 3.4 Profile Plot of Mean Knowledge Change Scores

To visualize the pattern reported in Section 3.3, mean change in knowledge score from baseline was plotted at each follow‐up wave (1‐month and 3‐month), separately by intervention arm and by intervention arm × gender. Because change scores are, by definition, zero at baseline, each profile begins at the origin and traces the average trajectory of knowledge gain or loss thereafter. Unlike the intention‐to‐treat model in Section3.3, the change scores plotted here are the observed (available‐case) values at each wave; cell sizes therefore vary slightly across time points, reflecting the pattern of loss to follow‐up already reported in Table 2.

As shown in Figure 3A, the web‐based arm exhibited a small, nonsignificant decline in mean knowledge from baseline to 1 month (*M* = −1.69 percentage points, 95% CI = [−4.72 to 1.35]), followed by a substantial increase by 3 months (*M* = 6.85, 95% CI = [3.77–9.92]). The conventional arm showed the mirror‐image pattern: a small, nonsignificant increase at 1 month (*M* = 0.51, 95% CI = [−0.34 to 1.35]) followed by a marked decline by 3 months (*M* = −5.18, 95% CI = [−8.66 to −1.70]). The point at which the two profiles cross, between the 1‐month and 3‐month assessments, provides a graphical illustration of the significant Arm × Time interaction reported in Section 3.3, *F*(2, 747) = 7.47, *p* = 0.001.

**Figure 3:**
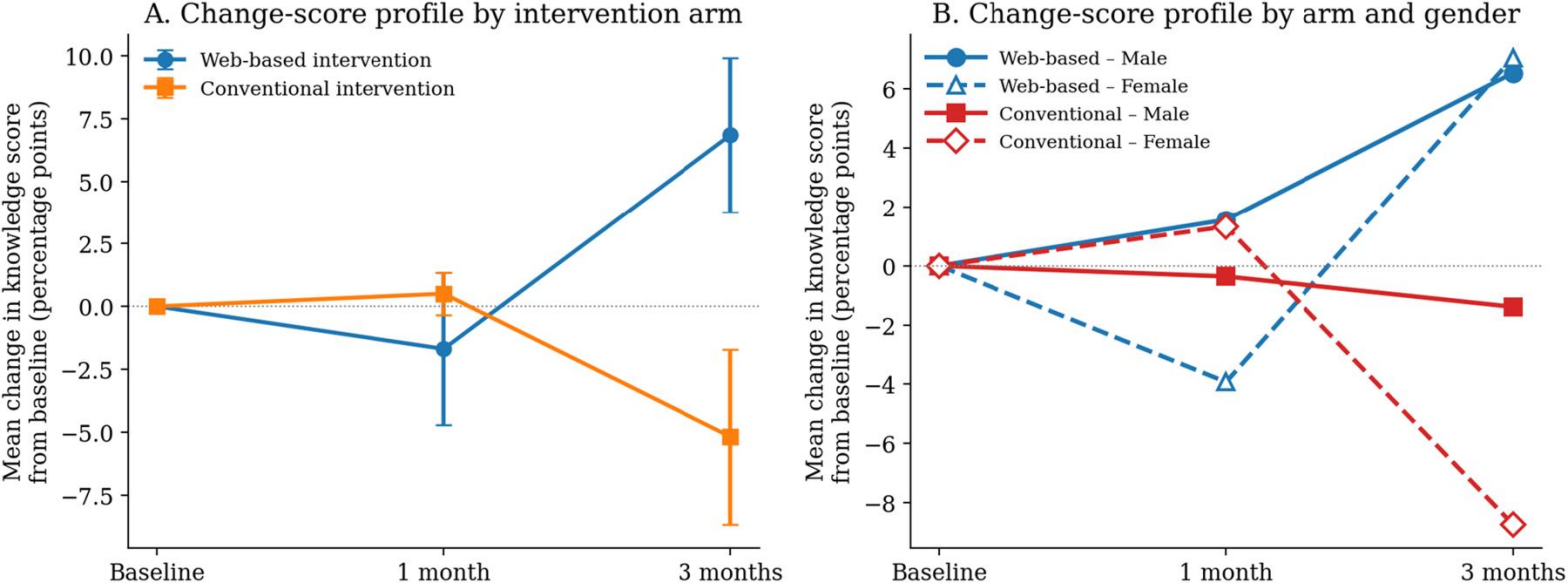
Profile plots of mean knowledge change score from baseline, by assessment wave. (A) By intervention arm. (B) By intervention arm and gender jointly. Error bars are 95% confidence intervals (CIs) (omitted in Panel B for legibility; see text). The dotted horizontal line marks no change from baseline. *Note:* Change scores (percentage points) are calculated as follow‐up minus baseline knowledge score, using observed cases available at each wave (1‐month: *n* = 130 web‐based, 125 conventional; 3‐month: *n* = 125 web‐based, 120 conventional; see Table 2 for full cell sizes). Values shown are unadjusted (i.e., not controlling for age), in contrast to the adjusted estimates from the repeated‐measures ANCOVA reported in Table 3; the two are consistent in direction and pattern. Formal significance tests of the group differences shown here (the Arm × Time and Arm × Gender × Time interactions, and their associated simple effects) are reported in Section 3.3 and Table 3, and are not repeated in this figure.

Figure 3 disaggregates this pattern by gender. The 3‐month gain in the web‐based arm was evident for both males (*M* = 6.54, 95% CI = [1.44–11.63]) and females (*M* = 7.06, 95% CI = [3.12–10.99]), despite females showing a transient decline at 1 month (*M* = −3.93, 95% CI = [−7.69 to −0.17]) not seen among males (*M* = 1.56, 95% CI = [−3.51 to 6.64]). In the conventional arm, the 3‐month decline was markedly steeper for females (*M* = −8.73, 95% CI = [−13.33 to −4.13]) than for males, whose change from baseline was small and not statistically distinguishable from zero (*M* = −1.39, 95% CI = [−6.61 to 3.83]). This visual pattern is consistent with, though not independently confirmed by, the marginal three‐way Arm × Gender × Time interaction reported in Section 3.3 (*p* = 0.053), and should be interpreted with the same caution.

## 4 Discussion

The current study provides evidence that a culturally focused, web‐based intervention is associated with better preservation of SRH knowledge over time than conventional face‐to‐face teaching among rural indigenous adolescents. Despite similar results for the two instructional modalities at baseline and 1 month after the intervention, a significant difference was observed at the 3‐month assessment. The web‐based cohort exhibited a consolidation effect, and knowledge scores continued to increase over time, whereas the conventional cohort showed significant knowledge decay. These results challenge the sufficiency of traditional school‐based lecturing in conservative environments and emphasize the centrality of digital privacy and gender relations in achieving long‐term educational retention.

The strongest finding was the large effect at the 3‐month time point (Cohen's *d* = 0.83; Table 2), reflected in a significant Arm × Time interaction (partial *η* ^2^ = 0.020; Table 3). This finding contrasts with previous meta‐analyses that argue digital interventions provide only temporary gains that disappear without reinforcement. Instead, our findings are in line with recent findings that mobile applications can generate self‐reinforcement (in a parallel fashion), allowing adolescents to secretly go through sensitive content and thus prevent the dramatic decays that have often been witnessed after one‐session workshops [2, 16, 17]. The sharp decrease in the conventional group, especially with female respondents, confirms the hypothesis of learning erosion, which suggests that when information is provided through didactic lectures in conservative societies, it quickly becomes forgotten because of the absence of follow‐up and the embarrassment related to the discussion of taboo topics in the open [14, 19].

A further contribution of this research is the descriptive pattern across the marginal Arm × Gender × Time interaction (*p* = 0.053; Table 3), which suggests digital learning may have been beneficial to all respondents, with a numerically larger benefit for females; this gender difference should be treated as exploratory rather than confirmed, since the two‐way Arm × Gender interaction was not itself significant (*p* = 0.167). Web‐based females showed the highest positive gain, which decreased slightly at 1 month and was highest at 3 months. This trend indicates that the anonymity of the mobile application was an effective control mechanism against the culture of silence and shame that unequally prevents indigenous girls from discussing SRH issues in face‐to‐face environments [4, 6]. On the other hand, the significant decline in knowledge among females in the conventional arm shows that traditional classroom settings can potentially contribute to the entrenchment of reticence or anxiety, thus hindering long‐term cognitive retention [1]. These results support the idea of digital tools as a safe space. Ippoliti and L'Engle [7] and Ssenfuka et al. [29] describe this. Digital platforms offer privacy. This privacy helps users avoid social stigma and judgment. For many young people, stigma gets in the way of seeking SRH information. This barrier tends to be worse for young women [30, 31]. Cartwright et al. [30] found that women in South Africa and Zambia valued self‐care options precisely because they avoided judgment from providers and community members. Timilsina et al. [31] reported similar findings among women of reproductive age more broadly. A private, digital format removes much of that risk.

The trend in the profile plots diverged: the web‐based arm showed a positive trend by 3 months, while the conventional arm showed a negative trend. This divergence is consistent with delivery mode differentially shaping the temporal trajectory of learning, though it may also reflect the difference in cumulative exposure between arms rather than delivery medium alone. The web‐based format presumably facilitated what educational theorists call spaced repetition, whereby the user voluntarily re‐read the application, which may have enhanced cognitive encoding [32]. Conversely, the conventional arm's trajectory is consistent with the limitations of a single‐dose model of education, which may not provide lasting reinforcement without follow‐up. The covariate effect of age was not significant, suggesting the observed pattern was broadly consistent across ages; however, internal validity remains constrained by the study's non‐randomized, two‐cluster design.

The study's strengths are its rigorous design, topicality, and accurate measures. The controlled longitudinal quasi‐experimental design with 1‐ and 3‐month follow‐ups strengthens the evidence base on the comparative effectiveness of web‐based versus conventional SRH education, although causal attribution is limited by the non‐randomized, two‐district allocation. The trial among rural Indigenous Bidayuh adolescents will fill a significant evidence gap for a poorly represented group widely used in digital SRH assessments. SRH modules and result tools have been systematically adapted, expert‐reviewed, and psychometrically validated, with high internal consistency, enhancing the reliability of the knowledge estimates. The study avoids a weakness found in prior studies by comparing two active interventions rather than an active arm against a no‐treatment control; however, because the two arms also differ in cumulative exposure (continuous digital access vs. a single session), the comparison reflects differences in dose as well as delivery modality. Lastly, temporal trends and subgroup effects can be examined more precisely through additional factorial ANCOVA with covariate adjustment and gender interaction testing, strengthening the research and making it more applicable to the problem at hand.

Several limitations should be considered when interpreting these findings. First, our primary analysis followed an intention‐to‐treat approach, analyzing all 260 enrolled participants according to their originally assigned condition using every assessment each participant provided; this reduces the risk of attrition bias relative to a complete‐case approach alone. We supplemented this with a per‐protocol (complete‐case) analysis and a multiple‐imputation sensitivity analysis, both of which produced estimates consistent with the intention‐to‐treat result. Nonetheless, non‐adherence and dropout due to technology constraints or SRH‐related stigma may still differ from real‐world implementation conditions, and future studies should continue to report and compare multiple analytic approaches to characterize this uncertainty. Second, this trial was conducted among Bidayuh adolescents from two districts in Sarawak, which limits the generalizability of the findings to other indigenous groups or urban youth. Beyond generalizability, because the intervention condition was assigned at the district level with only one district per arm, the effect of delivery modality cannot be statistically separated from unmeasured, time‐invariant differences between the two districts (e.g., local infrastructure, socioeconomic profile, or community attitudes toward SRH education); this is a structural limitation of the two‐cluster design that cannot be resolved through participant‐level statistical adjustment, and findings should be interpreted as hypothesis‐generating rather than definitive evidence of a causal effect of delivery modality. The intervention's effectiveness may also vary across settings with differing levels of digital literacy, internet connectivity, and cultural norms around SRH, so future studies in other rural and marginalized populations are needed before wider scale‐up. Third, although the app was culturally tailored and showed benefits for knowledge retention over 3 months, our trial did not evaluate how digital SRH interventions can be sustained or kept relevant over longer periods. In rural settings with fluctuating connectivity and device access, maintaining adolescents' engagement may be challenging, and additional strategies such as periodic content updates, reinforcement messages, integration with school or community programs, and offline or low‐bandwidth modules will be needed. Future implementation studies should explore these approaches to support long‐term use and scalability in diverse rural and marginalized populations. Finally, outcomes focused primarily on self‐administered knowledge measures rather than observed behaviors or clinical endpoints, limiting inferences about actual behavior change in SRH. Fourth, participants and facilitators were not blinded to allocation; therefore, expectancy or performance biases cannot be entirely ruled out, particularly for self‐report outcomes.

## 5 Conclusion

This longitudinal quasi‐experimental study suggests that a culturally adapted web‐based SRH intervention is associated with more durable knowledge gains than conventional classroom instruction among rural indigenous adolescents, with sustained improvement from 1 to 3 months in the digital arm, in contrast to knowledge decay in the conventional arm. The 3‐month benefit was numerically larger for female adolescents, who rebounded from an initial slight decline to achieve the highest 3‐month scores, though the three‐way Arm × Gender × Time interaction was only marginal (*p* = 0.053), and this gender difference should be treated as a descriptive pattern rather than a confirmed effect. Because the web‐based arm also received continuous access over 3 months compared with a single conventional session, and because the two arms were assigned by district rather than individually randomized, these findings cannot fully distinguish the effect of delivery mode from differences in cumulative exposure or from unmeasured district‐level factors; they should be interpreted as hypothesis‐generating rather than definitive evidence that delivery mode is the primary driver of learning consolidation. Future research should use individually randomized designs with matched exposure duration to test this more directly, examine whether retention translates into behavioral change, and evaluate strategies to extend engagement beyond 3 months to prevent decay in standard or minimally reinforced interventions. As a preliminary policy and practice implication, integrating confidential, self‐managed digital platforms into school and community SRH programs, complemented by periodic reinforcement mechanisms, may help enhance access and sustain learning outcomes in conservative rural communities, pending confirmation in a randomized, multi‐site trial.

## Author Contributions

**Md Mizanur Rahman:** conceptualization, methodology, supervision, formal analysis, validation, funding acquisition, writing – original draft, writing – review and editing, project administration. **Gerraint Gillan Ahi:** conceptualization, investigation, validation, formal analysis, project administration, writing – original draft, writing – review and editing, methodology. **Rafazila Ramli:** conceptualization, validation, formal analysis, investigation, writing – review and editing, methodology. **Rasitasam Safii:** conceptualization, writing – review and editing, supervision, methodology. **Jacey Lynn Minoi:** conceptualization, validation, methodology, writing – review and editing. **Stephanie Chua Hui Li:** conceptualization, writing – review and editing, methodology. **Ahmad Sofian bin Shminan:** conceptualization, writing – review and editing, methodology. **Lee Jun Choi:** conceptualization, writing – review and editing, methodology.

## Conflicts of Interest

The authors declare no conflicts of interest.

## Transparency Statement

The lead and Corresponding author, Md Mizanur Rahman, affirms that this manuscript is an honest, accurate, and transparent account of the study being reported; that no important aspects of the study have been omitted; and that any discrepancies from the study as planned have been explained.

## Data Availability

The data are not publicly available due to privacy or ethical restrictions. The data supporting this study's findings are available from the corresponding author upon request, provided that the request is made on reasonable grounds.
